# Do poorer people have poorer access to local resources and facilities? The distribution of local resources by area deprivation in Glasgow, Scotland^☆^

**DOI:** 10.1016/j.socscimed.2008.05.029

**Published:** 2008-09

**Authors:** Sally Macintyre, Laura Macdonald, Anne Ellaway

**Affiliations:** MRC Social and Public Health Sciences Unit, 4 Lilybank Gardens, Glasgow G12 8RZ, UK

**Keywords:** Scotland, Area deprivation, Location of resources, Deprivation amplification, Environmental justice, UK, Health inequalities

## Abstract

It has commonly been suggested that in modern cities individual or household deprivation (for example, low income or education) is amplified by area level deprivation (for example, lack of jobs or good schools), in ways which damage the health of the poorest and increase health inequalities. The aim of this study was to determine the location of a range of resources and exposures by deprivation in a UK city. We examined the location of 42 resources in Glasgow City, Scotland, in 2005–2006, by quintile of small area deprivation. Measures included number per 1000 population, network distance to nearest resource, and percentage of data zones containing at least one of each type of resource. Twelve resources had higher density in, and/or were closer to or more common in, more deprived neighbourhoods: public nurseries, public primary schools, police stations, pharmacies, credit unions, post offices, bus stops, bingo halls, public swimming pools, public sports centres, outdoor play areas, and vacant and derelict land/buildings. Sixteen had higher density in, and/or were closer to, or more common in, more affluent neighbourhoods: public secondary schools, private schools, banks, building societies, museums/art galleries, railway stations, subway stations, tennis courts, bowling greens, private health clubs, private swimming pools, colleges, A & E hospitals, parks, waste disposal sites, and tourist attractions. Private nurseries, Universities, fire stations, general, dental and ophthalmic practices, pawn brokers, ATMs, supermarkets, fast food chains, cafes, public libraries, golf courses, and cinemas showed no clear pattern by deprivation. Thus it appears that in the early 21st century access to resources does not always disadvantage poorer neighbourhoods in the UK. We conclude that we need to ensure that theories and policies are based on up-to-date and context-specific empirical evidence on the distribution of neighbourhood resources, and to engage in further research on interactions between individual and environmental factors in shaping health and health inequalities.

## Introduction

Public health reform movements were developed in many countries in the early 19th century in response to problems created by rapidly increasing urbanisation and industrialisation (Fee & Porter, 1992). Structured enquiries in a number of countries showed marked differences in health and life expectancy between social groups, between towns, and between neighbourhoods in towns. Edwin Chadwick, for example, noted striking differences between the age at death of ‘gentry and professionals’, ‘farmers and tradesmen’ and ‘labourers and artisans’, and also differences between less industrialised areas (for example, Bath and Rutland) and highly industrialised cities (such as Liverpool and Manchester) in the 1830s (Wohl, 1983). Farr, the British Registrar General, drew up life tables for ‘healthy districts’, which he used as a gold standard with which to contrast less salubrious areas and to argue that much premature mortality was due to environmental conditions and therefore preventable (Farr, 1975; Szreter, 1984).

The response of public health reformers was to tackle harmful aspects of the physical and social environment that most damaged the poor, by providing or legislating for clean water and air, drainage, sewerage, decent housing, education, and regulating working conditions (Fee & Porter, 1992). Indeed for about a century ‘public health’ was virtually synonymous with environmental improvements. However, with the decline in infectious diseases and the eradication of the most health damaging exposures, in the mid 12th century public health efforts were re-oriented towards a focus on individual risks and lifestyles contributing to the major chronic diseases. This policy shift was mirrored by a move away from ecological studies within epidemiology, towards an increased focus on individuals (Macintyre & Ellaway, 2000; Schwartz, 1994).

However, since the mid 1990s there has been renewed interest in the importance of environmental as well as individual characteristics in influencing health and health-related behaviours. An extensive literature has reviewed a distinction between compositional and contextual explanations for area variations in health (the former referring to characteristics of residents, the latter to characteristics of the area) (Diez-Roux, 1998, 2001; Kawachi & Berkman, 2003; Macintyre, Ellaway, & Cummins, 2002; Pickett & Pearl, 2001). Empirical studies have mostly found that who you are (e.g. age, gender, race, social class) is the strongest predictor of health and health-related behaviour, but that where you live also matters (Pickett & Pearl, 2001; Riva, Gauvin, & Barnett, 2007). This has been shown for total and coronary heart disease (CHD) mortality (Diez-Roux et al., 1997; Waitzman & Smith, 1998), CHD prevalence and risk factors (Davey Smith, Hart, Watt, Hole, & Hawthorne, 1998), morbidity (Jones & Duncan, 1995), depression (Yen & Kaplan, 1999b), diet, physical activity, smoking and alcohol consumption (Ecob & Macintyre, 2000; Ellaway & Macintyre, 1996; Karvonen & Rimpela, 1996).

The observation that health and health-related behaviours tend to be poorer in more disadvantaged areas, even after controlling for individual characteristics, has contributed to the re-emergence of the idea that, in general, environmental characteristics in poorer areas are detrimental to health and healthy living (Macintyre, Maciver, & Sooman, 1993). We have described this as ‘deprivation amplification’ (Macintyre, 2007), a pattern by which a range of resources and facilities which might promote health are less common in poorer areas (an extension of the ‘inverse care law’ first propounded in relation to health care (Tudor Hart, 1971)). A similar but converse idea is encapsulated in the notion of environmental injustice, which posits that environmental threats to health (e.g. waste disposal sites, air pollution, toxic factory fumes) are more likely to be located in poorer areas occupied by the least powerful in society (Hofrichter, 1993).

The deprivation amplification and environmental injustice concepts both imply that we might have to deal with possibly additive, interacting or multiplicative effects of personal and local resources (Gordon et al., 2003). Recent theorising on health and place has tended to argue that the compositional versus contextual distinction may be a false one, and that we need to study both structure and agency, and how people shape places as well as places shape people (Bernard et al., 2007; Cummins, Curtis, Diez-Roux, & Macintyre, 2007; Macintyre et al., 2002).

The research which suggested a residual influence of area of residence, after having controlled for individual characteristics, provided a useful impetus for empirical research into what features of areas might influence health and health-related behaviours, and for thinking about policies to improve environments. The general assumption in much recent literature is that the patterns of deprivation amplification and environmental injustice are common in modern cities, that is, that poorer neighbourhoods will usually have poorer access to health promoting resources and more exposure to health damaging ones, and that area deprivation thus typically compounds individual disadvantage.

For example, in relation to the location of retail stores in Canada, it has been stated:

‘Because low-income families have so little money to spend on any kind of product, their choices are restricted to one or two of the cheapest brands, or in some cases to the grim choice of doing without. Low income levels restrict shopping strategies as well. Without access to credit or savings, there is no possibility of stocking up on bargains or carrying over goods from one season to the next. Without a car, a poor or elderly household is at the mercy of the nearest retail outlets…Supermarkets in poor inner-city areas often stock poorer quality produce and meat, or charge higher prices, than suburban branches of the same chain. Merchants are not entirely to blame: they make their profit on large orders and expensive items; they lose money on the three dollar purchases. These differing expenditure profiles strongly affect the mix of stores in different types of neighbourhood. Well-to-do areas have many financial institutions, travel agencies and gift shops. Poorer districts have small food stores and personal services such as barber shops or shoe repair shops’ (Jones & Simmons, 1987) p. 45.

Pearce et al. describe as a common assumption:

‘that differential access to neighbourhood resources is one explanation for the observed gap in health between deprived and non-deprived neighbourhoods’ (Pearce, Witten, Hiscock, & Blakely, 2007) p. 349 and policy documents in the UK have emphasised the role of deprivation amplification in relation to several services and amenities, e.g.

‘those living in disadvantaged circumstances, who are most in need of the benefits of education, may be least able to gain access to them’ (Acheson, 1998) p. 38.

‘Many deprived areas lack core services, including education, health, financial and support. This is the result of a lack of targeted expenditure, difficulties in delivering services, and greater and more complex demands from residents of deprived areas. This lack increases the problems deprived neighbourhoods already face through restricting the opportunities available. Educational attainment, health, and quality of life are lowered, while unemployment and crime increase. This is now recognised as an important issue.’ (Renewal.net, 2007) p. 2.

It has been argued that racial residential segregation in the USA contributes to inequalities in health because it exposes black and ethnic minorities to poorer environments:

‘Segregation is a fundamental cause of differences in health status between African Americans and whites because it shapes socioeconomic conditions for blacks not only at the individual and household levels but also at neighbourhood and community levels. We review evidence that segregation…can create social and physical risks in residential environments that adversely affect health’ (Williams & Collins, 2001) p. 405.

For many researchers, activists and policy makers this emphasis on, or assumption of, deprivation amplification and/or environmental injustice is a welcome balance to the view that differences between areas are solely due to differences in the personal characteristics or behaviours of the residents. However, policies based on the deprivation amplification model may be misguided if based on poor empirical information (Macintyre, 2007). It may not always be true that poorer neighbourhoods are more likely to lack health promoting resources, and to be exposed to more health damaging resources. The spatial distribution of resources by deprivation may vary between types of resource, geographical location within a city, countries, and time periods.

Some empirical research has indeed found that the relationship between area deprivation and access to resources may vary by resource and national context. Studies of children's outdoor playgrounds have consistently found them to be more common in and closer to poorer areas (Cradock et al., 2005; Ellaway, Kirk, Macintyre, & Mutrie, 2007; Karsten, 2002; Smoyer-Tomic, Hewco, & Hodgson, 2004). However, other resources for physical activity may show a more varied pattern; e.g. in Perth, Australia, lower socio-economic status (SES) areas had better access to sports/recreation centres, gyms and swimming pools, while higher SES areas had better access to golf courses and the beach (Giles-Corti & Donovan, 2002); in Melbourne there were no differences in the number or total area of free access, restricted access or sporting/recreation open spaces by neighbourhood SES (Ball, Timperio, & Crawford, 2006); and a study in the Netherlands found there was no significant difference by neighbourhood SES in proximity to sports facilities (van Lenthe, Brug, & Mackenbach, 2005). However, a national study in the USA found that higher SES areas were better served with physical fitness facilities, membership sports and recreation clubs, dance facilities and public golf courses; these facilities were least likely to be present in areas with higher proportions of African American, Hispanic or other ethnic minority backgrounds (Powell, Slater, Chaloupka, & Harper, 2006).

In general, and consistently with the above analysis of racial residential segregation (Williams & Collins, 2001), recent studies in the USA are most likely to find that low SES and predominantly black areas lack services such as supermarkets (Chung & Myers, 1999; Morland, Wing, Diez Roux, & Poole, 2002; Zenk et al., 2005). These findings contrast with those outside the USA. For example, in Brisbane, Australia there were minimal or no socio-economic differences in food shopping infrastructure (Winkler, Turrell, & Patterson, 2006); in New Zealand travel distances to supermarkets were shorter in more deprived areas (Pearce, Blakely, Witten, & Bartie, 2007; Pearce, Witten et al., 2007); in the South East of the Netherlands there was increased proximity to food shops with increasing socio-economic disadvantage (van Lenthe et al., 2005), and in the UK there was no evidence of lack of access to supermarkets in poorer areas since changes in planning regulations in the 1990s (Competition Commission, 2000). It has been argued that there may be less evidence for the existence of ‘food deserts’ in the UK than is often supposed, although they may have existed a couple of decades ago (Cummins & Macintyre, 2002). A national study in New Zealand found that for 15 out of 16 measures of community resources, access was clearly better in more deprived neighbourhoods (Pearce, Witten et al., 2007).

However, even in the USA the pattern may be more complex than is often suggested, and patterns there may have changed over time. For example, in his classic 1977 book ‘Equality and Urban Policy; the Distribution of Municipal Public Services’, Lineberry tested three ‘underclass’ hypotheses about the distribution of resources in San Antonio, Texas: that the quantity and/or quality of urban services would be positively related to the proportion of anglos; higher SES people; and residents occupying positions of power in urban government. He examined the location and quality of parks, fire stations, libraries, and connection to water and sewers, and found that none of these hypotheses were supported: areas with more anglos, higher SES and members of power elites were further from parks, fire stations and libraries. The explanation was that older, denser neighbourhoods, more likely to be populated by poorer and ethnic minority residents, were closer to core public service facilities; residents of suburban areas had poorer access. An alternative, ‘ecological’ hypothesis (that it is attributes of neighbourhoods such as the age of the housing or population density which are related to service delivery) was supported. Lineberry noted that his observations in San Antonio were consistent with those in a range of other USA cities in the 1970s, including New York City, Detroit, Chicago, and Philadelphia, which mainly found either no clear relationship between race or income and facilities, or the location of facilities favouring low income areas.

A more extensive analysis was undertaken in Oklahoma City in the early 1980s, involving 17 facilities (private and public sector) across 184 census tracts characterised by a wide range of socio-economic variables. This found no straightforward relationship between poverty and access to resources, and instead identified seven different types of area differing in history and location, which had differential access to packages of resources; for example, one type of inner city area was characterised by good accessibility to employment and public parks but poor accessibility to community centres, libraries, hospitals and ambulance services; one type of area, forming a continuous ring round the inner city areas, had relatively high levels of income, education and property values but low accessibility to all the facilities examined and were ‘significantly deficient’ in access to several public services including elementary schools, post offices, libraries, and community centres (Knox, 1982).

Given such diverse findings, we decided to build on our previous work in Glasgow, Scotland, in which we had shown for two socially contrasting localities that many resources were more accessible and of better quality in the more socially advantaged area (Macintyre et al., 1993), by examining the location, by small area deprivation, of as wide a range of resources as we could find and geocode. The aim was to establish whether health promoting resources tended to be more available in richer areas, and potentially health damaging resources more common in poorer areas; and if not, whether there was any discernible pattern to the location, according to deprivation, of different types of resource.

## Design and methods

Starting from previous work in which we suggested using a framework of universal human needs which might be met by local opportunity structures (Macintyre et al., 1993, 2002), we first identified a number of high level domains (e.g. education, transport, health care) of services people need to live a healthy life. Within each of these domains we then searched for geocodable operationalisations (e.g. primary schools, secondary schools, bus stops, railway stations, general practices, opticians). As we have previously noted (Cummins, Macintyre, Davidson, & Ellaway, 2005), there is often a mismatch between the items one would ideally like to be able to collect and geocode, and those which are readily available in a reasonably reliable and up-to-date format. Here we report on 42 facilities/resources within the boundaries of Glasgow City Council in 2005–2006. (We investigated their distribution within the city boundaries only, as many of the resources examined are subject to local City Council planning decisions.) The numbers of each of these ranged from 4 (municipal waste disposal sites) to 3325 (bus stops) (see Table 1, first column, and web Table 1 in online version). Some items which might have been valuable in terms of models of pathways from the environment to health were not available for various reasons including a lack of any central register (e.g. tobacco sales points), the absence of suitably distributed measuring points (e.g. air quality), or lack of variation (e.g. all mains water in Glasgow comes from the same source).

Various sources were used to identify the location of these resources; most of the information was downloaded from the Internet (see web Table 2 in online version). The location was defined by unit postcode (zipcode; the smallest postal geography in the UK) or by *X* and *Y* co-ordinates.

Look-up tables were used to link the unit postcodes to Scottish data zones. The data zone is the key small area statistical geography in Scotland (Scottish Executive, 2004). The data-zone geography covers the whole of Scotland and nests within local government boundaries. Data zones are groups of 2001 Census output areas and the majority have populations of between 500 and 1000 residents. Where possible, they have been made to respect physical boundaries and natural communities. They have a regular shape and, as far as possible, contain households with similar social characteristics.

**Map 1:**
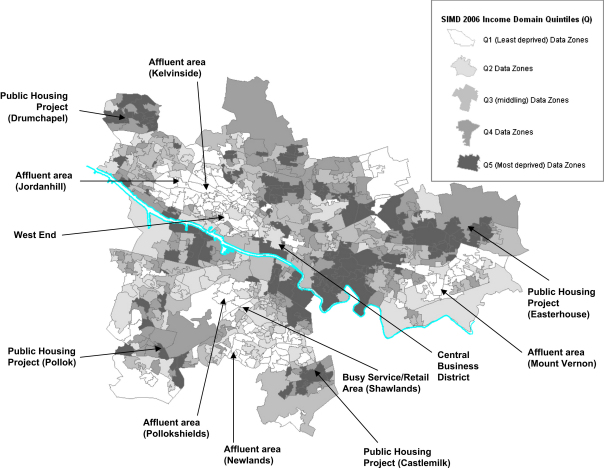
Glasgow City data zones by Scottish index of multiple deprivation 2006 income domain quintiles.

There are 694 data zones in the Glasgow City Council boundary (Map 1), with a mean population of 832 (range 248–2243) and a mean area of 25.2 ha (Scottish Executive, 2004). In 2004 Glasgow City had a population of around 577,670 people, and covered approximately 17,730 ha (Scottish Executive, 2004). For each data zone we obtained the 2006 Scottish Index of Multiple Deprivation (SIMD) Current Income sub-domain score (Scottish Executive, 2006). The SIMD is a publicly available continuous measure of compound social and material deprivation, calculated using data such as employment, welfare benefits, health, education and housing for each data zone. We chose not to use the full index since it includes health variables and access to services, so there might have been some circularity in investigating whether it predicted access to resources. The Current Income sub-domain is based on numbers of residents claiming a range of financial welfare benefits (e.g. Income Support, Guaranteed Pension Credit, Job Seekers Allowance (Scottish Executive, 2006)). We divided SIMD scores for Glasgow into quintiles (ranging from Quintile 1 = least deprived to Quintile 5 = most deprived). Quintiles 1–4 contain 139 data zones each while Quintile 5 contains 138 data zones. We calculated quintiles separately for the Glasgow City Council area (Map 1) (as opposed to using the existing Scotland wide categories) because deprived neighbourhoods are overrepresented in Glasgow if one uses the national classification. The average area of data zones differed slightly across these quintiles, being greatest (28.6 ha) in Quintile 4 and least (22.3 ha) in Quintile 5 (see web Table 3 in online version).

For 34 of the resources we used four measures of the distribution of resources in relation to deprivation: the percentage distribution across quintiles of each resource; the mean number of resources per 1000 population; the mean network distance in metres from the centroid of each data zone to the nearest of each of the resources; and the percentage of data zones containing at least one of each resource (see Table 1).

We used population data from the General Register for Scotland's 2004 small area estimates for each data zone (Scottish Executive, 2004) to calculate the density of each resource per 1000 people per quintile. (Areas without any resources were also included.) The analysis for nursery schools, primary and secondary schools was also repeated for density for 0–4 and 5–15 year olds only (Quintiles 4 and 5 had greater numbers of young people; see web Table 4 in online version). Comparison of density between quintiles was determined by ANOVA using SPSS version 14.0. We did not calculate mean numbers of Universities, colleges, A & E hospitals, waste disposal centres, cinemas and tourist attractions per 1000 people as there are fewer than 10 of each within Glasgow City. We did not calculate mean numbers per 1000 residents of parks or of vacant and derelict land/buildings (VDLB) as some of these cover more than one data zone.

Network analysis (i.e. finding the shortest path between two locations on a road network) was carried out for each resource (excluding parks and VDLB) using ArcGIS version 9.1. Street maps (including point addresses) were obtained from UK Ordinance Survey (Ordinance Survey, 2006). Every resource was geocoded by unit postcode and then matched to the street number and name. Network analysis was undertaken to find the network distance in metres from the centroid of each data zone to the nearest resource in each category (for example, nearest supermarket, sports centre, and primary school) and we then calculated the mean distance to the nearest resource within each SIMD quintile. Comparison between quintiles of mean distances to resources was determined by ANOVA in SPSS version 14.0.

Network analysis was not undertaken for parks as they cover more than a single point and this type of analysis cannot be carried out from a boundary. As an alternative, we created 500 m ‘service areas’ (i.e. polygons created around data-zone centroids using the distance travelled on a road as the edges) for each data zone, and noted whether or not there was a park within each service area. Comparison between quintiles in terms of percentages of data-zone service areas with parks was determined by Chi-Square. Network analysis was not carried out for VDLB as it seemed more appropriate to note its presence within a 500 m buffer from the data-zone centroid than calculate the network distance. Comparison between quintiles in terms of percentages of data-zone buffers with VDBL was determined by Chi-Square in SPSS 14.0.

The percentage distribution of each of the resources across the quintiles was calculated, and for each resource the percentage of data zones with at least one resource within each SIMD quintile was also calculated.

This was not a sample survey for which one would undertake power calculations to determine the achieved sample size that would be required to detect a given effect size at a conventional level of significance. As noted, the number of resources varied from 4 to 3325. Although we have tested the density and network distance measures for statistical significance (using the 0.05 level) the results should be treated with caution, and in interpreting the results we have not relied exclusively on statistical significance.

## Results

Within the education domain (see Table 1A), there was a stepwise association between the number of Local Education Authority (LEA) i.e. publicly funded, nurseries and primary schools per 1000 people and deprivation, with a higher density in poorer areas. There was no clear pattern of density by deprivation for LEA secondary schools, private nurseries or private schools. There were no significant differences between quintiles in mean numbers of nurseries per 1000 children under 5 years, and no significant differences for mean numbers of primary schools per 1000 children aged 5–11 years or secondary schools per 1000 children aged 11–16 years (tables not shown, available from authors). However, LEA nurseries and LEA primary schools were significantly closer to more deprived quintiles, while LEA secondary schools and private schools were significantly closer to more affluent quintiles. Private nurseries were fairly evenly distributed by deprivation.

There were no significant differences between quintiles in mean numbers per 1000 people of fire stations or police stations, although distance to the nearest police station was less for more deprived quintiles (and fire stations were slightly closer to more deprived quintiles) (see Table 1B). There were no significant differences in the density of GP surgeries, dental practices or ophthalmic practices by deprivation, but the most deprived quintile contained a significantly greater mean number of pharmacies. Distance to the nearest GP surgery was less for Quintiles 2 and 3, and there were no significant differences between quintiles in distances to the nearest dentist, pharmacy or ophthalmic practice (Table 1C). Quintiles of data zones did not differ in the proportions with at least one emergency or health service resource.

The mean number of credit unions and post offices per 1000 people rose with increasing deprivation (Table 1D). Although they did not show higher density per 1000 people, banks and building societies were significantly closer to Quintiles 1 and 2; and 36% banks, 58% of building societies, 40% pawn brokers/cheque cashers, and 35% ATMs were in Quintile 2. In contrast, as deprivation increased, distance to a credit union decreased, there were more per 1000 in poorer areas, and 34% of these were in Quintile 5. There were no significant differences between quintiles in density of or distance to the nearest pawn broker/cheque casher or ATM.

There were no significant associations between retail food outlets and deprivation. However, nearly a third of the fast food chains were located in Quintile 5, and a third of cafes were in Quintile 2 (Table 1E).

Public libraries were not significantly associated with deprivation (a third were in Quintile 3), bingo halls were more common in and closer to poorer areas, public museums/art galleries were closer to more affluent quintiles, and tourist attractions were closer to Quintiles 2 and 3 (Table 1F and Table 2).

Railway stations and subway stations were closer to more affluent areas, a relatively high proportion of these stations being located in Quintiles 2 and 3. Bus stops were significantly more prevalent in and closer to more deprived areas (Table 1G).

Public sports centres and children's play areas were more common in, and closer to, more deprived quintiles, and public swimming pools were closer to more deprived areas; in contrast, tennis courts and bowling clubs were more common in and closer to more affluent quintiles. Private health clubs and swimming pools were closer to more affluent areas (Table 1H).

There were no significant differences between quintiles in distance to the nearest University or Cinema (see Table 2). Colleges, A & E hospitals and tourist attractions were significantly closer to more affluent quintiles, but Quintile 2 showed the shortest distance to each of these. Waste disposal sites were closer to more affluent quintiles.

The proportion of data zones with VDLB increased in a stepwise manner with increasing deprivation, from 64% data zones with centroids within 500 m in Quintile 1 to 97% in Quintile 5 (see Table 3).

There were no significant differences between quintiles in the percentage with a public park within 500 m service areas of their centroids, although nearly 13% more of the data zones within QI were within this distance than Quintile 5 (see Table 4).

In summary of the 42 resources geocoded, 12 had higher density in, and/or were closer to or more common in, more deprived neighbourhoods: LEA nurseries, LEA primary schools, police stations, pharmacies, credit unions, post offices, bus stops, bingo halls, public swimming pools, public sports centres, outdoor play areas, and vacant and derelict land/buildings. Sixteen had higher density in, and/or were closer to, or more common in, more affluent neighbourhoods: LEA secondary schools, private schools, banks, building societies, museums/art galleries, railway stations, subway stations, tennis courts, bowling greens, private health clubs, private swimming pools, colleges, A & E hospitals, parks, waste disposal sites, and tourist attractions. Private nurseries, Universities, fire stations, general, dental and ophthalmic practices, pawn brokers, ATMs, supermarkets, fast food chains, cafes, public libraries, golf courses, and cinemas showed no clear pattern by deprivation.

## Discussion

Our findings on the distribution of resources in Glasgow City in 2005–2006 do not support a model of deprivation amplification, by which areas with poorer people are consistently more poorly served by public and private facilities. Rather they support a more differentiated model by which some resources are equally accessible to residents across a range of deprivation, some are more prevalent in and nearer to more affluent areas, and some are more prevalent in and nearer to more deprived areas.

Some resources appear to be located in ways which substitute or compensate for each other; for example, banks and building societies are commoner in more affluent areas and there has been a deliberate policy by local government to set up and support credit unions in poorer areas to help to compensate for the lack of banking facilities. Similarly, poorer areas have better access to public swimming pools and sports centres whereas richer areas have better access to private versions of these resources. Further work would be necessary to establish the historical sequence (many of the public sports resources in Glasgow are relatively old, compared to the recent development of chain private sports resources such as Next Generation, Virgin, Esporta), and also whether this substitution effect stems from market forces or from conscious planning decisions.

Many of the resources are most likely to be found in Quintile 2 (dental and ophthalmic practices, banks, building societies, pawn brokers, ATMs, cafes, museums/art galleries, railway and subway stations, private health clubs). This is probably because Quintile 2 is closer to the central business district and other retail, office and service hubs (e.g. the West End) than Quintile 1, which is more purely residential (see Map 1).

Despite what we described earlier as the common assumption that one reason for health differences between more and less deprived neighbourhoods is differences in access to resources, our findings are not inconsistent with evidence from a number of cities, in many countries, including a wide range of resources, and over a considerable period of time.

As noted in the Introduction, recent literature has similarly found that the relationship between area deprivation and access to resources may vary by the resource and national context in question.

It is likely that the patterns of racial residential segregation found in the USA may explain the differences between the USA and other nations in the extent of deprivation amplification in poor areas (Cummins & Macintyre, 2006; Williams & Collins, 2001), although the earlier USA studies cited in the Introduction still point to a more differentiated picture even in the USA (Knox, 1982; Lineberry, 1977).

However, there are a number of important caveats about our findings. Firstly, we measured availability of and distance to resources, and did not measure their quality. Although we have shown that poorer areas were not disadvantaged in terms of access to many of the resources mapped, this does not mean that they were not disadvantaged in terms of quality. For example, poorer communities had closer access to publicly funded primary schools and were not disadvantaged in access to secondary schools. However, Pacione has shown that in Glasgow the quality of publicly funded secondary schools was lower in poorer areas (Pacione, 1997). The issue of quality of services is one that requires more detailed analysis, some of which we are already undertaking (e.g. a study auditing the quality of children's playgrounds in the top, middle, and bottom quintiles of deprivation, and an analysis of the price and availability of a basket of grocery stores across Glasgow in 2007), and which is beyond the scope of this paper.

Secondly, much of the literature assumes that access to resources is health promoting. However, the Alameda County study in the USA found that higher rates per 1000 population of common commercial stores (including grocery stores, supermarkets, laundries/dry cleaners and pharmacies) predicted higher mortality (those who lived in neighbourhoods with many stores/services had a 32% increased risk of dying in the following 11 years, compared with people who lived in neighbourhoods with few stores) (Yen & Kaplan, 1999a). This suggests that proximity to some or many resources need not necessarily be health promoting. In much recent literature ‘fast food’ outlets tend to be treated as health damaging, and supermarkets as health promoting (Pearce, Blakely et al., 2007), and in the environmental justice literature proximity to facilities such as mobile phone masts, VDLB and waste dumps are treated as threats to wellbeing. However, we need to be more precise in theorising about, and finding empirical evidence for, any hypothesised links, whether positive or negative, with health. It is possible that some resources may be both health promoting and health damaging, or health promoting for some residents and health damaging for others; for example, proximity to a bus stop might be health promoting in facilitating access to employment or education and increasing levels of walking, but might be health damaging in producing diesel fumes, traffic noise, disturbance from passengers getting on or off buses, and pedestrian or cycling accidents. Vacant and derelict land may look threatening and stressful to some residents, but provide opportunities for outdoor physical activity for children or young males. Again, analysis of hypothesised pathways between access to resources and health requires more detailed study and is beyond the scope of this paper.

Thirdly, people may not use facilities in their immediate residential environment. It might be more important for some people to have a supermarket, pharmacy, bank, park, or sports centre near their place of work or their child's school. The relevance of local facilities may vary by stage in the life cycle, socio-economic status, gender (Kwan, 1999), car ownership, and other factors; for example, having a post office, supermarket and pharmacy very close might be much more important for a pensioner or non-employed single parent than for an employed person in his/her twenties. An ethnographic study of families in San Antonio, for example, found that more than 90% of 1000 destinations used were outside the census tract of residence. Those activities conducted closer to home were associated with food or grocery shopping, educational activities, recreation, health services and other services. Non-food shopping, work locations, and social support networks, were found in more distant locations (Matthews, Detwiler, & Burton, 2005).

A related issue is whether the location of facilities is designed to meet, or does meet, demand in the immediate area; such facilities may be targeted at and patronised by office workers, passing traffic, and users from other areas, and not seen by locals as being appropriate to them. A study in Glasgow of the opening of a supermarket in a poor neighbourhood found the main beneficiaries to have been people from outside the area who switched to that supermarket, rather than locals who continued to shop in smaller local shops and/or did not perceive the supermarket to be designed for them (Cummins et al., 2008; Cummins, Petticrew, Higgins, Findlay, & Sparks, 2005). Similarly, fast food outlets are often targeted not at those who live in close proximity, but at those working or shopping locally, using local bars or cinemas, or passing by (Macintyre, McKay, Cummins, & Burns, 2005; Schlosser, 2001). Residents of rich suburbs or gated communities may not wish to have commercial or publicly available facilities in their vicinity, and their greater ability to resist attempts to site such facilities near them may mean they are more likely to be located in less affluent localities.

Fourthly, there is the important issue of whether it is the objectively measured presence or absence of facilities that is most likely to influence behaviour, or the perceived or symbolic presence or absence of facilities. Evidence from the supermarket study shows that actual provision may not overcome symbolic barriers to use. Similarly we have found in Glasgow that answers to a question about whether a respondent lived within half a mile of a public green space did not show strong agreement with whether their home fell within a half mile buffer of a park; it seemed that some respondents did not feel the local park was culturally available or suitable for them (Macintyre, Ellaway, & McKay, 2006).

Fifthly, there is the issue of geographical scale. Here we have used relatively small areas which are designed to respect physical boundaries and natural communities and which are widely used in administrative geography. We have tried, by using network distances, to avoid the ‘container’ fallacy which appears to assume that all activities are contained within the boundaries of some administratively defined area; but the modifiable areal unit problem (MAUP) (Openshaw, 1984) may mean that we would have observed different results had we used larger, differently defined, types of area. Indeed, the different conclusions reached in this analysis using 694 small areas compared to those we have reached using two localities (pop size = c.50,000) or four neighbourhoods (pop size = c.25,000) in Glasgow may be a consequence of the MAUP. However, it is beyond the scope of this paper to re-examine our findings using different spatial scales, or to assess what would be appropriate scales for each resource. Our analysis also assumes that a similar friction of distance operates for all resources and social groups, which is unlikely (Handy & Niemeier, 1997).

Sixthly, our findings for particular resources can differ according to the measure used; one might reach slightly different conclusions if one focused on distance alone as compared with density per 1000 alone, or per total 1000 as compared with per 1000 of different ages (e.g. public primary schools). However, these differences are not substantial and do not alter the basic picture that there is not a clear, stepwise, relationship between affluence and resources across all exposures.

Despite the above caveats, our findings based on currently used administrative geography do not support a simple deprivation amplification model. Our findings support Lineberry's ‘ecological’ hypothesis that the location of urban resources are related to the age, history, geographical location, density, and residential/commercial mix of different areas, rather than his ‘underclass’ hypothesis (Lineberry, 1977). Many of our specific findings make sense in terms of the social, political and economic history of Glasgow and its socio-spatial development (for example, the construction of suburban railways by private companies, and municipal and philanthropic initiatives, in the 19th century; repeated initiatives for housing improvement including the post second world war dispersal of the poor to peripheral public housing estates, and the more recent gentrification of city centre and riverside areas (Map 1); and the history of migration from Ireland, Italy, and the Indian subcontinent (Pacione, 1995)). This suggests that we need to take a more nuanced, context- and resource-specific view of the distribution of resources by area deprivation, and of the relationship between services and facilities in an area and the health and health-related behaviours of its residents.

## Figures and Tables

**Table 1:** Per Scottish Index of Multiple Deprivation (SIMD) quintile: number of each resource; percentage of total resources; mean number per 1000 residents; mean distance to nearest; % data zones with at least one

	SIMD quintile	Number	Percentage of total resources	Mean *N* per 1000 residents	Mean distance (metres) to nearest resource	% DZs with at least one
*A. Education*						
*LEA nurseries*	1 – Most affluent	4	5.8	0.03	1292	2.9
	2	14	20.3	0.12	1079	10.1
	3 – Middling	12	17.4	0.10	1208	8.6
	4	15	21.7	0.13	1113	10.8
	5 – Most deprived	24	34.8	0.23	959	17.4
	Total	69	100.0	0.12	1131	9.9
	ANOVA			*F* = 5.09, *p* < 0.001	*F* = 5.11, *p* < 0.001	
	Linearity			*F* = 16.10, *p* < 0.001	*F* = 12.68, *p* < 0.001	

*Private nurseries*	1 – Most affluent	41	20.1	0.33	672	23.7
	2	35	17.2	0.30	709	23.0
	3 – Middling	39	19.1	0.31	745	19.4
	4	43	21.1	0.40	649	25.9
	5 – Most deprived	46	22.5	0.45	652	29.0
	Total	204	100.0	0.36	685	24.2
	ANOVA			*F* = 1.09, *p* = 0.362	*F* = 1.2, *p* = 0.345	
	Linearity			*F* = 3.07, *p* = 0.080	*F* = −0.67, *p* = 0.080	

*LEA primary schools*	1 – Most affluent	18	9.4	0.16	795	11.5
	2	34	17.7	0.28	624	23.0
	3 – Middling	42	21.9	0.35	600	26.6
	4	45	23.4	0.41	556	30.2
	5 – Most deprived	53	27.6	0.48	578	32.6
	Total	192	100.0	0.34	631	24.8
	ANOVA			*F* = 5.51, *p* < 0.001	*F* = 8.23, *p* < 0.001	
	Linearity			*F* = 21.52, *p* < 0.001	*F* = 22.84, *p* < 0.001	

*LEA secondary schools*	1 – Most affluent	9	30.0	0.07	1228	6.5
	2	5	16.7	0.04	1493	3.6
	3 – Middling	5	16.7	0.04	1459	3.6
	4	8	26.7	0.08	1585	5.8
	5 – Most deprived	3	10.0	0.02	1662	2.2
	Total	30	100.0	0.05	1485	4.3
	ANOVA			*F* = 1.37, *p* = 0.242	*F* = 6.82, *p* < 0.001	
	Linearity			*F* = 1.02, *p* = 0.313	*F* = 23.2, *p* < 0.001	

*Private schools*	1 – Most affluent	4	33.3	0.03	2270	2.9
	2	4	33.3	0.03	2372	2.9
	3 – Middling	3	25.0	0.03	2846	1.4
	4	1	8.3	0.01	3291	0.7
	5 – Most deprived	0	0.0	0.00	3390	0.0
	Total	12	100.0	0.02	2833	1.6
	ANOVA			*F* = 1.03, *p* = 0.389	*F* = 16.14, *p* < 0.001	
	Linearity			*F* = 3.50, *p* = 0.062	*F* = 61.38, *p* < 0.001	

*B. Emergency services*						
*Fire stations*	1 – Most affluent	0	0.0	0.00	2437	0.0
	2	4	30.8	0.04	2278	2.9
	3 – Middling	2	15.4	0.02	2173	1.4
	4	2	15.4	0.02	2180	1.4
	5 – Most deprived	5	38.5	0.04	2091	2.9
	Total	13	100.0	0.02	2232	1.7
	ANOVA			*F* = 1.06, *p* = 0.374	*F* = 25, *p* = 0.062	
	Linearity			*F* = 1.62, *p* = 0.204	*F* = 8.01, *p* = 0.005	

*Police stations*	1 – Most affluent	1	4.5	0.01	1832	0.7
	2	5	22.7	0.05	1585	3.6
	3 – Middling	6	27.3	0.04	1621	4.3
	4	4	18.2	0.04	1621	2.9
	5 – Most deprived	6	27.3	0.05	1518	4.3
	Total	22	100.0	0.04	1636	3.2
	ANOVA			*F* = 0.91, *p* = 0.458	*F* = 2.28, *p* = 0.024	
	Linearity			*F* = 1.67, *p* = 0.198	*F* = 7.14, *p* = 0.008	

*C. Health services*						
*GP surgeries*	1 – Most affluent	17	16.2	0.15	1013	10.8
	2	18	17.1	0.15	825	11.5
	3 – Middling	34	32.4	0.27	814	19.4
	4	18	17.1	0.17	853	12.2
	5 – Most deprived	18	17.1	0.17	913	12.3
	Total	105	100.0	0.18	884	13.3
	ANOVA			*F* = 1.51, *p* = 0.197	*F* = 2.86, *p* = 0.023	
	Linearity			*F* = 0.14, *p* = 0.713	*F* = 1.26, *p* = 0.262	

*Dental practices*	1 – Most affluent	25	18.7	0.20	872	13.7
	2	38	28.4	0.30	831	21.6
	3 – Middling	28	20.9	0.25	896	15.8
	4	18	13.4	0.16	927	11.5
	5 – Most deprived	25	18.7	0.22	929	14.5
	Total	134	100.0	0.23	891	15.4
	ANOVA			*F* = 1.16, *p* = 0.328	*F* = 0.77, *p* = 0.547	
	Linearity			*F* = 0.56, *p* = 0.456	*F* = 1.99, *p* = 0.159	

*Pharmacies*	1 – Most affluent	19	12.1	0.17	806	12.2
	2	32	20.4	0.28	706	20.9
	3 – Middling	35	22.3	0.30	689	20.1
	4	27	17.2	0.24	727	15.8
	5 – Most deprived	44	28.0	0.40	716	23.9
	Total	157	100.0	0.28	729	18.6
	ANOVA			*F* = 2.43, *p* = 0.046	*F* = 1.35, *p* = 0.252	
	Linearity			*F* = 6.12, *p* = 0.014	*F* = 1.63, *p* = 0.203	

*Ophthalmic practices*	1 – Most affluent	14	13.7	0.12	1156	9.4
	2	32	31.4	0.27	1036	13.7
	3 – Middling	18	17.6	0.16	1136	11.5
	4	10	9.8	0.09	1184	5.0
	5 – Most deprived	28	27.5	0.23	1150	11.6
	Total	102	100.0	0.17	1132	10.2
	ANOVA			*F* = 1.79, *p* = 0.130	*F* = 0.81, *p* = 0.521	
	Linearity			*F* = 0.03, *p* = 0.872	*F* = 0.47, *p* = 0.494	

*D. Means of exchange*						
*Banks*	1 – Most affluent	20	18.2	0.18	1085	10.8
	2	40	36.4	0.32	1080	7.9
	3 – Middling	23	20.9	0.20	1223	10.1
	4	7	6.4	0.06	1345	4.3
	5 – Most deprived	20	18.2	0.17	1340	8.0
	Total	110	100.0	0.18	1214	8.2
	ANOVA			*F* = 0.91, *p* = 0.457	*F* = 3.62, *p* = 0.006	
	Linearity			*F* = 0.87, *p* = 0.351	*F* = 12.83, *p* < 0.001	

*Building societies*	1 – Most affluent	2	16.7	0.02	3318	1.4
	2	7	58.3	0.06	3204	0.7
	3 – Middling	2	16.7	0.01	3815	0.7
	4	0	0.0	0.00	4240	0.0
	5 – Most deprived	1	8.3	0.01	4496	0.7
	Total	12	100.0	0.02	3814	0.7
	ANOVA			*F* = 0.680, *p* = 0.606	*F* = 8.51, *p* < 0.001	
	Linearity			*F* = 0.791, *p* = 0.374	*F* = 30.93, *p* < 0.001	

*Credit unions*	1 – Most affluent	0	0.0	0.00	2112	0.0
	2	4	11.4	0.03	2121	2.9
	3 – Middling	9	25.7	0.07	1565	6.5
	4	10	28.6	0.09	1471	7.2
	5 – Most deprived	12	34.3	0.10	1290	8.0
	Total	35	100.0	0.06	1712	4.9
	ANOVA			*F* = 3.74, *p* = 0.005	*F* = 21.59, *p* < 0.001	
	Linearity			*F* = 14.51, *p* < 0.001	*F* = 77.67, *p* < 0.001	

*Pawn brokers/cheque cashers*	1 – Most affluent	1	4.0	0.01	2414	0.7
	2	10	40.0	0.08	2286	2.9
	3 – Middling	4	16.0	0.04	2339	2.9
	4	3	12.0	0.03	2535	1.4
	5 – Most deprived	7	28.0	0.06	2491	4.3
	Total	25	100.0	0.04	2413	2.4
	ANOVA			*F* = 0.79, *p* = 0.530	*F* = 0.63, *p* = 0.639	
	Linearity			*F* = 0.16, *p* = 0.689	*F* = 0.97, *p* = 0.326	

*Post offices*	1 – Most affluent	13	12.7	0.11	791	9.4
	2	16	15.7	0.13	786	11.5
	3 – Middling	22	21.6	0.20	705	15.1
	4	19	18.6	0.16	755	13.7
	5 – Most deprived	32	31.4	0.30	761	21.7
	Total	102	100.0	0.18	760	14.3
	ANOVA			*F* = 3.86, *p* = 0.004	*F* = 0.87, *p* = 0.481	
	Linearity			*F* = 11.63, *p* = 0.001	*F* = 0.62, *p* = 0.430	

*ATMs*	1 – Most affluent	96	15.0	0.76	532.0	36.7
	2	215	33.5	1.74	481.1	45.3
	3 – Middling	122	19.0	1.00	561.4	37.4
	4	79	12.3	0.70	550.2	37.4
	5 – Most deprived	129	20.1	1.19	538.9	31.2
	Total	641	100.0	1.08	532.7	37.6
	ANOVA			*F* = 1.11, *p* = 0.348	*F* = 0.939, *p* = 0.440	
	Linearity			*F* = 0.024, *p* = 0.877	*F* = 0.674, *p* = 0.412	

*E. Food retail*						
*Supermarkets*	1 – Most affluent	11	24.4	0.09	1166	7.9
	2	6	13.3	0.05	1205	2.2
	3 – Middling	12	26.7	0.11	1280	7.9
	4	5	11.1	0.05	1304	3.6
	5 – Most deprived	11	24.4	0.11	1369	7.2
	Total	45	100.0	0.08	1265	5.8
	ANOVA			*F* = 0.87, *p* = 0.481	*F* = 2.26, *p* = 0.061	
	Linearity			*F* = 0.04, *p* = 0.850	*F* = 8.89, *p* = 0.003	

*Fast food chains*	1 – Most affluent	9	21.4	0.07	1800	4.3
	2	9	21.4	0.07	1659	2.2
	3 – Middling	7	16.7	0.06	1715	4.3
	4	4	9.5	0.04	1887	2.2
	5 – Most deprived	13	31.0	0.11	1745	5.1
	Total	42	100.0	0.07	1761	3.6
	ANOVA			*F* = 0.452, *p* = 0.771	*F* = 1.22, *p* = 0.303	
	Linearity			*F* = 0.158, *p* = 0.691	*F* = 0.22, *p* = 0.636	

*Cafes*	1 – Most affluent	52	16.5	0.41	917.9	21.6
	2	101	32.1	0.86	722.9	31.7
	3 – Middling	65	20.6	0.53	830.0	23.0
	4	33	10.5	0.31	909.8	15.8
	5 – Most deprived	64	20.3	0.55	1001.7	21.7
	Total	315	100.0	0.53	876.3	22.8
	ANOVA			*F* = 1.51, *p* = 0.197	*F* = 3.19, *p* < 0.05	
	Linearity			*F* = 0.286, *p* = 0.593	*F* = 3.60, *p* = 0.058	

*F. Culture and entertainment*						
*Bingo halls*	1 – Most affluent	1	7.7	0.01	2887	0.7
	2	1	7.7	0.01	2699	0.7
	3 – Middling	3	23.1	0.02	2553	2.2
	4	3	23.1	0.03	2337	2.2
	5 – Most deprived	5	38.5	0.04	2223	3.6
	Total	13	100.0	0.02	2540	1.9
	ANOVA			*F* = 1.12, *p* = 0.345	*F* = 5.55, *p* < 0.001	
	Linearity			*F* = 4.26, *p* = 0.040	*F* = 22.06, *p* < 0.001	

*Public libraries*	1 – Most affluent	4	11.1	0.05	1384	2.2
	2	5	13.9	0.04	1232	3.6
	3 – Middling	12	33.3	0.11	1174	8.6
	4	7	19.4	0.06	1191	5.0
	5 – Most deprived	8	22.2	0.07	1193	5.8
	Total	36	100.0	0.07	1235	5.0
	ANOVA			*F* = 1.01, *p* = 0.402	*F* = 2.20, *p* = 0.068	
	Linearity			*F* = 0.62, *p* = 0.431	*F* = 5.33, *p* = 0.021	

*Public museums/art galleries*	1 – Most affluent	0	0.0	0.00	3318	0.0
	2	7	43.8	0.06	3162	2.9
	3 – Middling	3	18.8	0.02	3706	2.2
	4	5	31.3	0.04	3948	2.2
	5 – Most deprived	1	6.3	0.01	4132	0.7
	Total	16	100.0	0.03	3652	1.6
	ANOVA			*F* = 1.61, *p* = 0.169	*F* = 4.99, *p* = 0.001	
	Linearity			*F* = 0.00, *p* = 0.973	*F* = 17.30, *p* < 0.001	

*G. Transport*						
*Railway stations*	1 – Most affluent	14	24.1	0.11	1264	9.4
	2	17	29.3	0.14	1069	10.8
	3 – Middling	14	24.1	0.11	1195	9.4
	4	6	10.3	0.05	1421	4.3
	5 – Most deprived	7	12.1	0.06	1561	4.3
	Total	58	100.0	0.09	1302	7.6
	ANOVA			*F* = 1.63, *p* = 0.165	*F* = 8.39, *p* < 0.001	
	Linearity			*F* = 4.34, *p* = 0.038	*F* = 20.16, *p* < 0.001	

*Subway stations*	1 – Most affluent	2	13.3	0.01	3986	1.4
	2	5	33.3	0.05	3597	3.6
	3 – Middling	4	26.7	0.03	3891	2.2
	4	1	6.7	0.01	4341	0.7
	5 – Most deprived	3	20.0	0.02	4648	2.2
	Total	15	100.0	0.02	4092	2.0
	ANOVA			*F* = 1.19, *p* = 0.312	*F* = 3.98, *p* = 0.003	
	Linearity			*F* = 0.25, *p* = 0.619	*F* = 10.17, *p* = 0.001	

*Bus stops*	1 – Most affluent	482	14.5	3.89	306	82.0
	2	640	19.2	5.57	244	88.5
	3 – Middling	705	21.2	5.93	215	92.8
	4	665	20.0	5.97	191	92.1
	5 – Most deprived	833	25.1	7.53	234	96.4
	Total	3325	100.0	5.78	238	90.3
	ANOVA			*F* = 6.0, *p* < 0.001	*F* = 7.1, *p* < 0.001	
	Linearity			*F* = 20.9, *p* < 0.001	*F* = 14.9, *p* < 0.001	

*H. Physical activity and sport*						
*Public swimming pools*	1 – Most affluent	1	8.3	0.00	2675	0.7
	2	1	8.3	0.01	2387	0.7
	3 – Middling	2	16.7	0.02	2101	1.4
	4	5	41.6	0.05	1978	3.6
	5 – Most deprived	3	25.0	0.02	1899	2.2
	Total	12	100.0	0.02	2208	1.7
	ANOVA			*F* = 1.26, *p* = 0.284	*F* = 13.5, *p* < 0.001	
	Linearity			*F* = 2.21, *p* = 0.137	*F* = 50.7, *p* < 0.001	

*Private swimming pools*	1 – Most affluent	3	16.7	0.03	3372	2.2
	2	6	33.3	0.05	3353	2.9
	3 – Middling	3	16.7	0.03	3339	1.4
	4	0	0.0	0.00	3905	0.0
	5 – Most deprived	6	33.3	0.04	3681	1.4
	Total	18	100.0	0.03	3530	1.6
	ANOVA			*F* = 0.65, *p* = 0.627	*F* = 2.82, *p* = 0.024	
	Linearity			*F* = 0.13, *p* = 0.724	*F* = 6.04, *p* = 0.014	

*Public sports centres*	1 – Most affluent	2	6.9	0.01	1877	1.4
	2	1	3.4	0.01	1706	0.7
	3 – Middling	8	27.6	0.07	1539	5.8
	4	10	34.5	0.09	1525	7.2
	5 – Most deprived	8	27.6	0.07	1532	5.1
	Total	29	100.0	0.05	1636	4.0
	ANOVA			*F* = 2.64, *p* = 0.033	*F* = 4.57, *p* = 0.001	
	Linearity			*F* = 7.32, *p* = 0.007	*F* = 14.52, *p* < 0.001	

*Private health clubs*	1 – Most affluent	3	13.0	0.03	2517	2.2
	2	9	39.1	0.07	2576	4.3
	3 – Middling	5	21.7	0.04	2742	3.6
	4	1	4.3	0.01	3196	0.7
	5 – Most deprived	5	21.7	0.04	2915	2.9
	Total	23	100.0	0.04	2789	2.7
	ANOVA			*F* = 1.07, *p* = 0.369	*F* = 3.35, *p* = 0.010	
	Linearity			*F* = 0.54, *p* = 0.462	*F* = 8.87, *p* = 0.003	

*Tennis courts*	1 – Most affluent	11	57.9	0.08	1938	7.9
	2	5	26.3	0.04	2152	3.6
	3 – Middling	2	10.5	0.01	2847	1.4
	4	1	5.3	0.01	3178	0.7
	5 – Most deprived	0	0.0	0.00	3230	0.0
	Total	19	100.0	0.03	2668	2.7
	ANOVA			*F* = 4.52, *p* = 0.001	*F* = 19.97, *p* < 0.001	
	Linearity			*F* = 15.72, *p* < 0.001	*F* = 73.98, *p* < 0.001	

*Bowling clubs*	1 – Most affluent	11	22.4	0.08	1125	7.9
	2	16	32.7	0.14	1096	10.1
	3 – Middling	14	28.6	0.11	1293	9.4
	4	5	10.2	0.05	1418	3.6
	5 – Most deprived	3	6.1	0.03	1678	2.2
	Total	49	100.0	0.08	1322	6.6
	ANOVA			*F* = 2.81, *p* = 0.025	*F* = 13.28, *p* < 0.001	
	Linearity			*F* = 5.19, *p* = 0.023	*F* = 47.76, *p* < 0.001	

*Golf courses*	1 – Most affluent	4	40.0	0.04	2830	2.9
	2	0	0.0	0.00	2849	0.0
	3 – Middling	2	20.0	0.02	2659	1.4
	4	3	30.0	0.02	2727	1.4
	5 – Most deprived	1	10.0	0.01	3082	0.7
	Total	10	100.0	0.02	2829	1.3
	ANOVA			*F* = 1.11, *p* = 0.350	*F* = 2.32, *p* = 0.056	
	Linearity			*F* = 0.69, *p* = 0.405	*F* = 1.30, *p* = 0.255	

*Public play areas*	1 – Most affluent	72	12.6	0.67	628	21.6
	2	98	17.2	0.83	563	26.6
	3 – Middling	141	24.7	1.10	503	36.7
	4	112	19.6	0.97	487	44.6
	5 – Most deprived	148	25.9	1.37	451	49.3
	Total	571	100.0	0.99	527	35.7
	ANOVA			*F* = 2.73, *p* = 0.028	*F* = 5.03, *p* = 0.001	
	Linearity			*F* = 9.05, *p* = 0.003	*F* = 19.09, *p* < 0.001	

**Table 2:** For resources with <10 in Glasgow: mean distance in metres to nearest resource per SIMD quintile

	FE college	University	A & E hospital	Cinema	Tourist attraction	Waste disposal site
SIMD quintile						
1 Most affluent	3121	4756	3436	3890	4083	3523
2	2635	4839	3268	3990	3589	3703
3 Middling	2973	4816	3716	3909	3928	3796
4	3328	5159	4021	4260	4204	4336
5 Most deprived	3660	5165	4422	4182	4572	3879

Total	3143	4947	3772	4046	4074	3847

ANOVA	*F* = 5.5, *p* < 0.001	*F* = 0.9, *p* = 0.452	*F* = 6.7, *p* < 0.001	*F* = 0.9, *p* < 0.473	*F* = 3.4, *p* = 0.009	*F* = 5.1, *p* < 0.001
Linearity	*F* = 11.8, *p* < 0.001	*F* = 3.0, *p* = 0.083	*F* = 23.3, *p* < 0.001	*F* = 2.3, *p* = 0.127	*F* = 6.7, *p* = 0.010	*F* = 9.9, *p* = 0.002

**Table 3:** Percentage of data zones with vacant or derelict land/building (VDLB) within a 500 m buffer of their centroids by SIMD quintile

	Within 500 m of VDLB		Not within 500 m of VDLB		Total	
	*N*	%	*N*	%	*N*	%
SIMD quintile						
1 Most affluent	89	64.0	50	36.0	139	100.0
2	115	82.7	24	17.3	139	100.0
3 Middling	120	86.3	19	13.7	139	100.0
4	132	95.0	7	5.0	139	100.0
5 Most deprived	134	97.1	4	2.9	138	100.0

Total	590	85.0	104	15.0	694	100.0

Chi-Square	Value = 75.43, *p* = 0.000					

**Table 4:** Percentage of data zones with city, district or local parks within 500 m of their centroids by SIMD quintile

	Within 500 m of a green space		Not within 500 m of green space		Total	
	*N*	%	*N*	%	*N*	%
SIMD quintile						
1 Most affluent	65	46.8	74	53.2	139	100.0
2	52	37.4	87	62.6	139	100.0
3 Middling	57	41.0	82	59.0	139	100.0
4	57	41.0	82	59.0	139	100.0
5 Most deprived	47	34.1	91	65.9	138	100.0

Total	278	40.1	416	59.9	694	100.0

Chi-Square	Value = 5.18, *p* = 0.269					
