# Supplementary material for: Do poorer people have poorer access to local resources and facilities? The distribution of local resources by area deprivation in Glasgow, Scotland
Source: Soc Sci Med. 2008 Sep;67(6):900–14. doi: 10.1016/j.socscimed.2008.05.029 (PMC2570170; doi:10.1016/j.socscimed.2008.05.029)
Supplement: Supplementary file 1 [file mmc1.doc]

**Web Table 1 Number of resources identified**

|  | ***N*** |
| --- | --- |
| **Education** |  |
| LEA Nurseries | 69 |
| Private Nurseries | 204 |
| LEA Primary Schools | 192 |
| LEA Secondary Schools | 30 |
| Private Schools | 12 |
| Colleges | 9 |
| Universities | 5 |
|  |  |
| **Emergency Services** |  |
| A & E hospitals | 5 |
| Fire Stations | 13 |
| Police Stations | 22 |
|  |  |
| **Health** |  |
| GP Surgeries | 105 |
| Dental Practices | 134 |
| Pharmacies | 157 |
| Ophthalmic Practices | 102 |
|  |  |
| **Means of Exchange** |  |
| Banks | 110 |
| Building Societies | 12 |
| Credit Unions | 35 |
| Pawn Brokers/Cheque Cashers | 25 |
| Post Offices | 102 |
| ATMs | 641 |
|  |  |
| **Food Retail** |  |
| Supermarkets | 45 |
| Fast food chain restaurants | 42 |
| Cafes | 315 |
|  |  |

|  | ***N*** |
| --- | --- |
| **Culture and Entertainment** |  |
| Cinemas | 5 |
| Bingo Halls | 13 |
| Tourist Attractions | 8 |
| Public Libraries | 36 |
| Public Museums/Art Galleries | 16 |
|  |  |
| **Transport** |  |
| Railway Stations | 58 |
| Subway Stations | 15 |
| Bus Stops | 3325 |
|  |  |
| **Physical Activity and Sport** |  |
| Public Swimming Pools | 12 |
| Private Swimming Pools | 18 |
| Public Sports Centres | 29 |
| Private Health Clubs | 23 |
| Tennis Courts | 19 |
| Bowling Clubs | 49 |
| Golf Courses | 10 |
| Public Play Areas | 571 |
|  |  |
| **Land use** |  |
| Vacant and Derelict Land/Building | 852 |
| District, City or Local Parks | 77 |
| Waste Disposal Site | 4 |
|  |  |
|  |  |
|  |  |

**Web Table 2 Sources of data for each resource**

| **Resource** | **Source** | **Accessed** |
| --- | --- | --- |
| **Education** |  |  |
| Nurseries | Glasgow City Council (http://www.glasgow.gov.uk/) | July 2005 |
| LEA Primary Schools | http://www.glasgow.gov.uk/ | July 2005 |
| LEA Secondary Schools | http://www.glasgow.gov.uk/ | July 2005 |
| Private Nurseries | HM Inspectorate (http://www.hmie.gov.uk/) | Feb 2006 |
| Private Schools | Good Schools Guide (http://www.goodschoolsguide.com) | July 2005 |
| Universities | Online yellow pages (http://www.yell.co.uk) | Feb 2006 |
| FE Colleges | Glasgow Colleges Group (http://www.glasgowcolleges.ac.uk) | Feb 2006 |
| **Emergency Services** |  |  |
| A & E Hospitals | NHS Scotland (http://www.show.scot.nhs.uk/) | Dec 2005 |
| Police Stations | Strathclyde Police (http://www.strathclyde.police.uk) | Dec 2005 |
| Fire Stations | Strathclyde Fire & Rescue (http://www.strathclydefire.org) | Dec 2005 |
| **Health** |  |  |
| GP Surgeries | NHS Scotland | Aug 2005 |
| Dental Practices | Dental Directorate, Glasgow. | Aug 2005 |
| Pharmacies | Greater Glasgow Primary Care NHS Trust | Aug 2005 |
| Ophthalmic Practices | NHS Scotland | Aug 2005 |
| **Means of Exchange** |  |  |
| Banks | www.cbonline.co.uk, www.abbey.co.uk, www.bankofscotland.co.uk, www.rbs.co.uk, www.lloydstsb.com, www.alliance-leicester.co.uk www.bbg.co.uk, www.natwest.com, www.barclays.co.uk, www.aibgb.co.uk | Aug 2006 |
| Building Societies | www.nationwide.co.uk, www.dunfermline.com, www.ybs.co.uk, www.skipton.co.uk, www.britannia.co.uk, www.scottishbldgsoc.co.uk, www.leeds-holbeck.co.uk | Aug 2006 |
| Credit Unions | Association of British Credit Unions Ltd (www.abcul.coop/page/index.cfm) | Aug 2006 |
| Pawn Brokers/Cheque Cashers | http://www.yell.co.uk | Sept 2005 |
| Post Offices | http://www.yell.co.uk | Aug 2006 |
| ATMs | http://www.link.co.uk |  |
| **Food Retail** |  |  |
| Supermarkets | Glasgow City Council Environmental Health Dept, http://www.sainsbury.co.uk, http://www.morrisons.co.uk,  http://www.asda.co.uk, http://www.tesco.com  http://uk.aldi.com, http://www.farmfoods.co.uk/, http://www.iceland.co.uk/, http://www.lidl.co.uk | Aug 2006 |
| Fast food chains | http://www.yell.co.uk, http://www.burgerking.co.uk,  http://www.pizzahut.co.uk, http://www.mcdonalds.co.uk | Aug 2006 |
| Cafes | Glasgow City Council Environmental Health Dept |  |
| **Culture and Entertainment** |  |  |
| Cinemas | http://www.yell.co.uk, http://www.cineworld.co.uk, http://www.odeon.co.uk | Aug 2006 |
| Bingo Halls | http://www.glasgow.gov.uk/ | Aug 2005 |
| Tourist Attractions | http://www.glasgow.gov.uk/ | Aug 2006 |
| Public Museums/Art Galleries | http://www.glasgow.gov.uk/ | Aug 2006 |
| Public Libraries | http://www.glasgow.gov.uk/ | Aug 2005 |
|  |  |  |
| **Physical Activity and Sport** |  |  |
| Public Swimming Pools | http://www.glasgow.gov.uk/ | June 2005 |
| Private Swimming Pools | http://www.thefitmap.co.uk/healthclubs/results.htm  http://www.yell.co.uk | Oct 2006 |
| Public Sports Centres | http://www.glasgow.gov.uk/ | June 2005 |
| Private Health Clubs | http://www.yell.co.uk | Sept 2006 |
| Tennis Courts | http://www.glasgow.gov.uk/, http://www.yell.co.uk | Aug 2006 |
| Bowling Clubs | http://www.glasgow.gov.uk/, http://www.yell.co.uk | June 2005 |
| Golf Courses | http://www.glasgow.gov.uk/ | June 2005 |
| Public Play Areas | http://www.glasgow.gov.uk/ | April 2006 |
| **Transport** |  |  |
| Railway Stations | http://edina.ac.uk/digimap/index.shtml, www.yell.co.uk | Nov 2005 |
| Subway Stations | http://www.spt.co.uk | Feb 2006 |
| Bus Stops | National Public Transport Access Node database http://www.naptan.org.uk/ | April 2006 |
| **Land Use** |  |  |
| Vacant & Derelict Land/Buildings | GCC: Development and Regeneration Services | Dec 2005 |
| District, City or Local Parks | http://www.glasgow.gov.uk/ | Oct 2006 |
| Waste disposal sites | http://www.glasgow.gov.uk/ | Feb 2006 |

**Web Table 3 Mean size (Hectares) of data zones within each SIMD quintile in Glasgow**

|  | ***N*** | ***Mean Ha*** |
| --- | --- | --- |
| **SIMD Quintile** |  |  |
| **1 Most Affluent** | 139 | 24.6 |
| **2** | 139 | 24.5 |
| **3 Middling** | 139 | 26.0 |
| **4** | 139 | 28.6 |
| **5 Most Deprived** | 138 | 22.3 |
|  |  |  |
| **Total** | 694 | 25.2 |

**Web Table 4** **SIMD Current Income sub-domain Quintiles: Age-groups, 2004**

|  | ***Aged 0 to 4*** | | ***Aged 5 to 15*** | | ***Aged 16 to 64*** | | ***Aged 65 plus*** | | ***Total*** | |
| --- | --- | --- | --- | --- | --- | --- | --- | --- | --- | --- |
| ***SIMD Quintile** | **%** | **N** | **%** | **N** | **%** | **N** | **%** | **N** | **%** | **N** |
| **1 Most Affluent** | 5.03 | 6094 | 11.50 | 13937 | 71.65 | 86851 | 11.83 | 14337 | 100.00 | 121219 |
| **2** | 4.84 | 5603 | 10.54 | 12192 | 71.31 | 82467 | 13.30 | 15383 | 100.00 | 115645 |
| **3 Middling** | 4.43 | 5207 | 10.63 | 12493 | 66.63 | 78306 | 18.30 | 21509 | 100.00 | 117515 |
| **4** | 5.39 | 6192 | 12.66 | 14546 | 64.40 | 74005 | 17.56 | 20178 | 100.00 | 114921 |
| **5 Most Deprived** | 6.58 | 7132 | 14.86 | 16103 | 64.12 | 69486 | 14.44 | 15649 | 100.00 | 108370 |
| Total | 5.23 | 30228 | 11.99 | 69271 | 67.71 | 391115 | 15.07 | 87056 | 100.00 | 577670 |

*SIMD Quintiles 1-4 include 139 Data zones while Quintile 5 includes 138 Data zones each.
